# Palliation of Bone Cancer Pain by Antagonists of Platelet-Activating Factor Receptors

**DOI:** 10.1371/journal.pone.0091746

**Published:** 2014-03-17

**Authors:** Katsuya Morita, Seiji Shiraishi, Naoyo Motoyama, Tomoya Kitayama, Takashi Kanematsu, Yasuhito Uezono, Toshihiro Dohi

**Affiliations:** 1 Department of Pharmacology, Faculty of Nursing, Hiroshima Bunka Gakuen University, Hiroshima, Japan; 2 Cancer Pathophysiology, Division National Cancer Center Research Institute, Tokyo, Japan; 3 Department of Dental Science for Health Promotion, Division of Integrated Health Sciences, Institute of Biomedical and Health Sciences, Hiroshima University, Hiroshima, Japan; 4 Department of Cell and Molecular Pharmacology, Division of Basic Life Science, Institute of Biomedical and Health Sciences, Hiroshima University, Hiroshima, Japan; 5 Department of Pharmacotherapy, Pharmaceutical Sciences, Nihon Pharmaceutical University, Saitama, Japan; University of Arizona, United States of America

## Abstract

Bone cancer pain is the most severe among cancer pain and is often resistant to current analgesics. Thus, the development of novel analgesics effective at treating bone cancer pain are desired. Platelet-activating factor (PAF) receptor antagonists were recently demonstrated to have effective pain relieving effects on neuropathic pain in several animal models. The present study examined the pain relieving effect of PAF receptor antagonists on bone cancer pain using the femur bone cancer (FBC) model in mice. Animals were injected with osteolytic NCTC2472 cells into the tibia, and subsequently the effects of PAF receptor antagonists on pain behaviors were evaluated. Chemical structurally different type of antagonists, TCV-309, BN 50739 and WEB 2086 ameliorated the allodynia and improved pain behaviors such as guarding behavior and limb-use abnormalities in FBC model mice. The pain relieving effects of these antagonists were achieved with low doses and were long lasting. Blockade of spinal PAF receptors by intrathecal injection of TCV-309 and WEB 2086 or knockdown of the expression of spinal PAF receptor protein by intrathecal transfer of PAF receptor siRNA also produced a pain relieving effect. The amount of an inducible PAF synthesis enzyme, lysophosphatidylcholine acyltransferase 2 (LPCAT2) protein significantly increased in the spinal cord after transplantation of NCTC 2472 tumor cells into mouse tibia. The combination of morphine with PAF receptor antagonists develops marked enhancement of the analgesic effect against bone cancer pain without affecting morphine-induced constipation. Repeated administration of TCV-309 suppressed the appearance of pain behaviors and prolonged survival of FBC mice. The present results suggest that PAF receptor antagonists in combination with, or without, opioids may represent a new strategy for the treatment of persistent bone cancer pain and improve the quality of life of patients.

## Introduction

Pain in cancer is produced by pressure on, or chemical stimulation of, specialized pain-signalling nerve endings called nociceptors (nociceptive pain) or it may be caused by damage or illness affecting nerve fibers themselves (neuropathic pain) which is responsive to stimuli that are normally non-painful; allodynia. Bone cancer pain is one of the most common and usually serious pain conditions in cancer patients [1].

Opioids remain the mainstay of cancer pain management, but in addition to the acute side effects the long-term consequences of tolerance, dependency, hyperalgesia and the suppression of the hypothalamic/pituitary axis should be acknowledged. Because the current available treatments are relatively ineffective against bone cancer pain, almost half of cancer patients have inadequate pain control [2], [3]. Thus, the development of novel analgesics effective at treating bone cancer pain are required.

We have previously suggested that platelet-activating factor (PAF) may be a mediator of neuropathic pain. PAF injection into the mouse spinal cord caused thermal hyperalgesia and tactile allodynia, which were at least in part mediated by spinal dysfunction of glycine receptor α3 (GlyRα3) and were blocked by PAF receptor antagonists [4], [5]. Subsequent studies showed PAF receptor blockade reduced pain behaviors elicited in nerve injury models. A PAF receptor antagonist, CV-3988, injected near the dorsal root ganglion (DRG) in rats or mice lacking PAF receptors showed a reduction in tactile allodynia following spinal nerve injury [6]. Intrathecal injection of the PAF receptor antagonist, WEB 2086, a benzodiazepine derivative for 9 days post-surgery in rats suppressed the development of mechanical allodynia in a rat spared nerve injury model [7]. PAF receptor antagonists, TCV-309 (PAF related), BN 50739 (natural product related compound), and WEB 2086, produced profound and long lasting anti-allodynia effects in several different neuropathic pain models in mice, including a partial sciatic nerve ligation injury model, a partial infraorbital nerve ligation model, a chronic constriction of the infraorbital nerve injury model (CCI model) and a streptozotocin (STZ)-induced diabetes model [8]. The evidence suggests that PAF contributes to neural tissue damage and pain behavior after nerve injury.

The animal models above investigated pain due to bone cancer, in which tumor cells are injected locally into the bone. The present study used the femur bone cancer (FBC) model in mice [9]. Animals were injected with osteolytic NCTC2472 cells, and subsequently the effects of PAF receptor antagonists on pain behaviors were evaluated.

## Materials and Methods

### Experimental Animals

The experiments were performed using male C3H/HeN mice (CLEA Japan, Inc., Tokyo), weighing 20–25 g. All experimental procedures and animal handling were performed according to both the Guiding Principles for the Care and Use of Laboratory Animals approved by the Japanese Pharmacological Society and the guidelines of Hiroshima University, Hiroshima, Japan and the guidelines of National Cancer Center Research Institute, Tokyo, Japan. The protocol was approved by the committee on the Ethics of Animal Experiments of the Hiroshima University (Permit Number: A-09-17 and A-11-16), and of the National Cancer Center (Permit Number: T11-004-M02). All surgery was performed under sodium pentobarbital anesthesia, and all efforts were made to minimize suffering. The animals were used for only one measurement in each experiment.

### The Mouse Femur Bone Cancer (FBC) Model

For the FBC model, NCTC 2472 tumor cells (American Type Cuiture Collection, ATCC; Manassas, VA, USA) were injected into the medullary cavity of the distal femur of C3H/HeN mice [9]. The NCTC 2472 cells were maintained in Dulbecco's Modified Eagle's Medium, supplemented with 10% Fetal bovine serum, 100 unit/ml penicillin, and 100 μg/ml streptomycin (all products from Gibco Laboratories); and cultured at 37°C in a humidified atmosphere of 5% CO_2_ then passaged weekly according to ATCC guidelines. For administration, cells were detached by scraping and then centrifuged at 900 rpm for 3 min. The pellet was suspended in Hank's balanced salt solution (HBSS) and then used for intratibial injection.

For implantation, tumor cells were injected following the protocol described previously by Honore et al. [9] with slight modification. In brief, C3H/HeN mice were anesthetized with a sodium pentobarbital (60 mg/kg) i.p. injected. For intratibial inoculation of cells, the right knee of each mouse was bent and placed facing the experimenter and a minimal skin incision was made exposing the tibial plateau. A 25 gauge dental reamer was used to perforate the tibial plateau and, once removed, a 30 gauge needle coupled to a Hamilton syringe filled with the cell suspension was carefully introduced into the medullary cavity of the tibia. Then, 10^5^ NCTC 2472 cells suspended in 5 μl of HBSS were slowly injected (living cells). Control groups were injected with 5 μl of HBSS or HBSS containing 10^5^ NCTC 2472 cells killed by quickly freezing and thawing them twice without cryoprotection and then washing with fresh HBSS (deaden cells). Finally, Caviton EX, a dental-grade hydraulic temporary sealant (GC Dental Products, Co. Ltd., Tokyo, Japan) was applied to the tibial plateau incised area and the surgical procedure was completed with stitching of the knee skin. Body weights were recorded for each mouse every 3 days throughout the study. The animals were euthanized following weight loss greater than 20% of body weight or upon demonstrating related symptoms such as serious ataxic movements or other neurological abnormalities due to ethical concerns.

### Drug Administration

Gabapentin (1-(aminomethyl)-cyclohexaneacetic acid) was obtained from Sigma-Aldrich (St. Louis, MO). Morphine was obtained from Takeda Pharmaceutical Co., (Osaka, Japan). WEB 2086 (3-[4-(2-chlorophenyl)-9- methyl-6*H*-thieno[3,2-*f*][1], [2], [4]triazolo-[4,3-*a*][1], [4]-diazepin-2-yl]-1-(4-morpholinyl)-1-propanone) was obtained from Tocris Bioscience (Ellisville, MO). TCV-309 (3-bromo-5-[*N*-phenyl-*N*-[2-[[2-(1,2,3,4-tetrahydro-2-isoquinolyl- carbonyloxy)ethyl]carbamoyl]ethyl]carbamoyl]-1-propylpyridinium nitrate), and BN 50739 (tetrahydro-4,7,8,10methyl-(chloro-2phenyl)6[dimethoxy-3,4- phenylthio]methylthiocarbonyl-9 pyrido[4′,3′-4,5]thieno[3,2-*f*]triazolo-1,2,4[4,3-*a*]diazepine-1,4) were donated from Takeda Pharmaceutical Co., and Institute Henri Beaufour, respectively.

TCV-309 and WEB 2086 were each dissolved in sterile artificial cerebrospinal fluid (ACSF) or saline solution. BN 50739 was dissolved in a solvent containing 25% 2-hydroxypropyl-β-cyclodextrin (Sigma/RBI, Natick, MA) and distilled water, pH adjusted to ∼6 using 1 N NaOH, and diluted appropriately with ACSF or saline. Other reagents were dissolved in ACSF or saline. The drug solutions were freshly prepared on each experimental day. ACSF composition (in mM) was NaCl 142, KCl 5, CaCl_2_ 2H_2_O 2, MgCl_2_·6H_2_O 2, NaH_2_PO_4_ 1.25, D-glucose 10, HEPES 10, pH 7.4. TCV-309, BN70329, or WEB 2086 was administered intravenously 30 min-3 hr before pain assessment.

### Knockdown of PAF Receptor in the Spinal Cord

Knockdown of PAF receptor was performed according to a previous report [5]. siRNAs were incorporated into the hemagglutinating virus of the Japan (HVJ)-Envelope Vector according to the manufacturer's instructions. Briefly, after mixing 40 μl (1 assay unit, AU) of HVJ-Envelope Vector with 4 μl of the enclosing factor, the mixture was centrifuged (10,000×*g*, 10 min, 4°C), and the pellet suspended in 10 μl of buffer solution. Then, 10 μl of a mixture of 3 siRNAs solution (#1, #2 and #3, 1 μg/μl each) was added, and the mixture was kept on ice for 5 min. Sterile ACSF (10 μl) containing synthetic siRNA duplexes (0.45 pmole/animal) was injected into the subarachnoid space between the L5 and L6 vertebrates of conscious mice. The sequences of the siRNA oligonucleotide (sense) were as follows: PAF receptor (#1, 5′-CACCUCAGUGAGAAGUUUUACAGCA-AG-3′; #2, 5′-ACCCUUCCAAGAAACUAAAUGAGAU-AG-3′; #3, 5′-CAACUUCCAUCAGGCUAUUAAUGAU-AG-3′; targeting sequences around position 1005 to 1029, 232 to 256, 893 to 917, respectively, in *pafr*, GenBank accession no. D5087). Moreover, mismatched siRNA with three or four nucleotide mismatches was prepared to examine nonspecific effects of siRNA duplexes (siRNA#4, 5′-ACUCUGCCAAGAGACUACAUGAGAU-AG-3). These selected sequences were also submitted to a BLAST search (Bioinformatics Center Institute for Chemical Research, Kyoto University, Japan) against the mouse genome sequence to ensure that only one gene in the mouse genome was targeted. siRNAs were purchased from iGENE Therapeutics Inc. (Tsukuba, Japan).

### Behavioral Analysis in the FBC Model

The behavioral analysis was preformed following the protocol described previously [5], [10]. The pain-related behaviors in the FBC model were evaluated before and after drug administration on 11 days after tumor implantation. The experimental and sham operated animals were evaluated for on-going pain based on guarding behavior, for ambulatory pain based on limb-use abnormality, and for allodynia-like behavior based on the von Frey filament test and paintbrush test. Guarding behavior, limb-use abnormality and allodynia-like pain were assessed in the same animals. The mice were placed in a clear plastic observation box and allowed to habituate for 15 min. Then, spontaneous guarding behavior was assessed during a 2-min observation period. The lifting time of the hind paw on the ipsilateral side during ambulation was measured as guarding behavior. Limb-use abnormality was scored on a scale of 0 to 4: 0, normal use of limb; 1, slight limp; 2, clear limp; 3, partial non-use of limb; and 4, complete non-use of limb. Allodynia-like behavior was assessed by lightly stroking the injured leg with a paintbrush (allodynia score) or evaluated by measuring the paw withdrawal threshold in response to probing with a series of calibrated fine filaments (allodynia threshold) as reported previously [5]. The allodynia score was ranked as described by Minami et al. [11]: 0, no response; 1, mild squeaking with attempts to move away from the stroking probe; 2, vigorous squeaking, biting the stroking probe and strong efforts to escape from the stroking probe. Values are the average of the total score evaluated 3 times at each time point (possible maximum score at each time point: 2/mouse). The guard times were significantly prolonged at 5 and 6 days after tumor implantation compared with the values at pre-implantation. Similarly, animals also started to exhibit abnormal limb-use at 5 days after tumor implantation, and such abnormal behavior was more prominent in the later days. The allodynia threshold was evaluated by measuring the paw withdrawal threshold in response to probing with von Frey monofilaments, and significant threshold declines were observed after the 3^rd^ day post tumor implantation compared with the values at pre-implantation, and the decrease reached a peak after 5 and 7 days. The allodynia score was evaluated by measuring the ranked response to lightly stroking with a paintbrush, and significant score rises were observed after the 4-day post tumor implantation compared with the values at pre-implantation, and the increase reached a peak after 6 and 8 days. Throughout the experiment, behavioral testing was performed under blind conditions by a single experimenter. Injections were performed blind by another person. Intrathecal injection was performed as described previously [4].

### Determination of Lyso-PAF-Acetyltransferase (Lyso-PAF AT)/Lysophospha- tidylcholine Acyltransferase 2 (LPCAT2), a PAF Synthesis Enzyme

The lumbar regions of the spinal cord (L2-L6) were obtained from sham-operated (deaden cells implanted) or tumor-bearing mice. The spinal cord tissues (4.8–5.0 mg) were homogenized in 20 mM Tris-HCl (pH 7.5) containing 1 mM EDTA, 1 mM EGTA, 10 mM NaF, 10 mM β-glycerophosphate and 1 μg/ml of various protease inhibitors (benzamdine, leupeptin and antipain). The homogenate was reacted with 0.6% NP-40 for 5 min at 4°C, followed by centrifugation at 15,000 rpm for 5 min at 4°C and the supernatant was obtained. The supernatant was mixed at a volume ration of 4∶1 in 10 mM Tris-HCl (pH 6.8) containing 10% (v/v) glycerol, 2% sodium dodecylsulfate (SDS), 0.01% bromophenol blue and 0.5% β-mercaptoethanol, with subsequent boiling at 70°C for 30 min.

### Immunoblotting Assays

The obtained samples were electrophoresed for 2 hr at room temperature on polyacrylamide gels (stacking gel 3% and separating gel 10%) and then blotted to polyvinylidene fluoride membranes previously treated with 100% methanol. The transferred proteins on the membranes were blocked by incubating with blocking solution, a 5% skim milk-containing wash buffer (20 mM Tris-HCl (pH 7.5), 137 mM NaCl and 0.1% Tween 20), at 25°C for 1 hr. The membranes were exposed to the primary antibody as follows: against LPCAT2 (1∶1,000; Sigm); against β-actin (1∶1,000; IMGENEX Innovations in Functional Genomics, SanDiego, CA) diluted with the wash buffer containing 1% skim milk overnight at 4°C, followed by washing three times with the wash buffer, and then incubation with a secondary antibody as follows: for the LPCAT2 antibody and β-actin antibody, an anti-rabbit IgG antibody conjugated with horseradish peroxidase (1∶2,000; DakoCytomation, Glostrup, Denmark) at room temperature for 1 hr. The membranes were again rinsed three times with the wash buffer and developed with an enhanced chemiluminescence (ECL) western detection system (ECL reagents, purchased from Thermo Fisher Scientific, and Nacalai Tesque Inc., Kyoto Japan; ImageQuant™ LAS 4000 mini detection system, GE healthcare Japan, Tokyo, Japan). The density of each band was analyzed using NIH Image software, and the densitometric units were corrected using the value of β-actin. The data are expressed as the mean ± SEM.

### Morphine-induced Constipation

Mice were provided food and water ad libitum before the test period, and both (with or without TCV-309) groups consumed comparable amounts of food prior to the test as measured over a 24-hr period in the test environment. No food or water was available during the test. Mice were caged in acrylic boxes with grid floors suspended over filter paper. Fecal boli were collected from each group of mice every hour for 1 hr following the injection of saline or various doses of morphine and the weight of fecal boli was recorded as an index of the constipating effects of morphine.

### Generation of Kaplan–Meier Survival Curves

The health of the mice was examined daily. Mice that presented with signs of ill health were killed by cervical dislocation according to the recommendations of our Ethical Committee. In our experience, these signs precede the time of death by a couple of days. The time of death was recorded for each mouse that was found dead or was killed. Kaplan–Meier survival curves were generated using Graphpad Prism software (version 4; Graphpad, Inc, San Diego, CA).

### Statistical Analyses

All data are reported as values of the mean ± SEM (standard error of the mean). Graphpad Prism software version 4 was used to perform the statistical analysis. Regarding the statistical analysis of the pain-related behaviors, comparisons of the allodynis score, withdrawal threshold, guarding behavior and limb-use abnormality of the differences between the drug-treated groups and the vehicle-treated group were evaluated using two-way ANOVA followed by Tukey-Kramer test, or an unpaired Student's *t*-test. Comparisons of median survival were performed using log-rank and Gehan–Breslow–Wilcoxon tests. A probability value (P) of <0.05 was considered statistically significant.

## Results

### Effects of Systemic Administration of Specific PAF Receptor Antagonists on Tactile Allodynia and Pain Behaviors in FBC Model Mice

**Figure 1:**
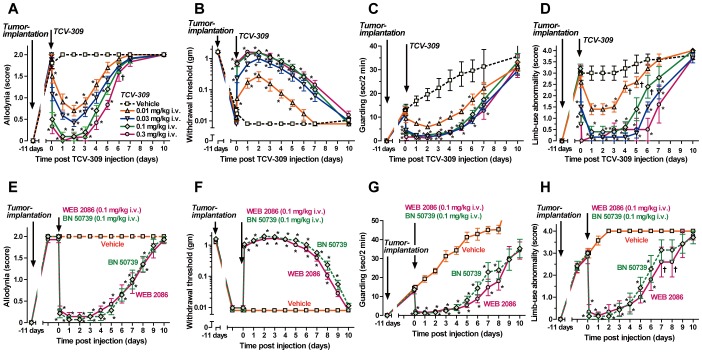
Effects of PAF-receptor antagonists on tactile allodynia (A, B, E, F), guarding behavior (C, G) and limb-use abnormality (D, H) in the femur bone cancer (FBC) mice. TCV-309 0.01-0.3 mg/kg, WEB 2086 0.1 mg/kg, BN 50739 0.1 mg/kg or a vehicle were intravenously injected at 11 days post tumor transplantation. Control mice received injections with a vehicle: saline or 25% 2-hydroxypropyl-β-cyclodextrin. The pain-related behaviors that developed after tumor implantation in mice were not affected by vehicle treatments. Values represent the mean ± SEM. n = 11 mice per group. †P<0.05, *P<0.01 compared with the corresponding control (vehicle treated) values, as determined by analysis of variance followed by Tukey-Kramer test.

Effects of TCV-309 on allodynia and pain behaviors (guarding behavior and limb-use abnormality) were examined in FBC model mice. TCV-309 with a range of 10–300 μg/kg by i.v. dose-dependently reduced the allodynia score (Figure 1A) and increased the withdrawal threshold (Figure 1B) at 11 to 15 days after tumor implantation in the mice. The effects of TCV-309 were quite long lasting. For instance, TCV-309 significantly ameliorated mechanical allodynia by a single injection of 100 μg/kg, i.v. with a peak effect at 1 to 2 days after the injection up to 6 days. Guarding behavior was continuously aggravated during the observation period, while limb-use abnormality reached a maximal response and continued during the period (Figure 1C, 1D). Guarding behavior and limb-use abnormality were also improved by TCV-309. TCV-309 up to 1 mg/kg did not affect general behavior or motor function estimated by the RotaRod test (data not shown). Other PAF receptor antagonists, BN 50739 and WEB 2086, also ameliorated the allodynia and pain behaviors in the FBC model mice (Figure1E–1H).

### Effects of Blockade of Spinal PAF Receptors on Allodynia and Pain Behaviors in FBC Model Mice

**Figure 2:**
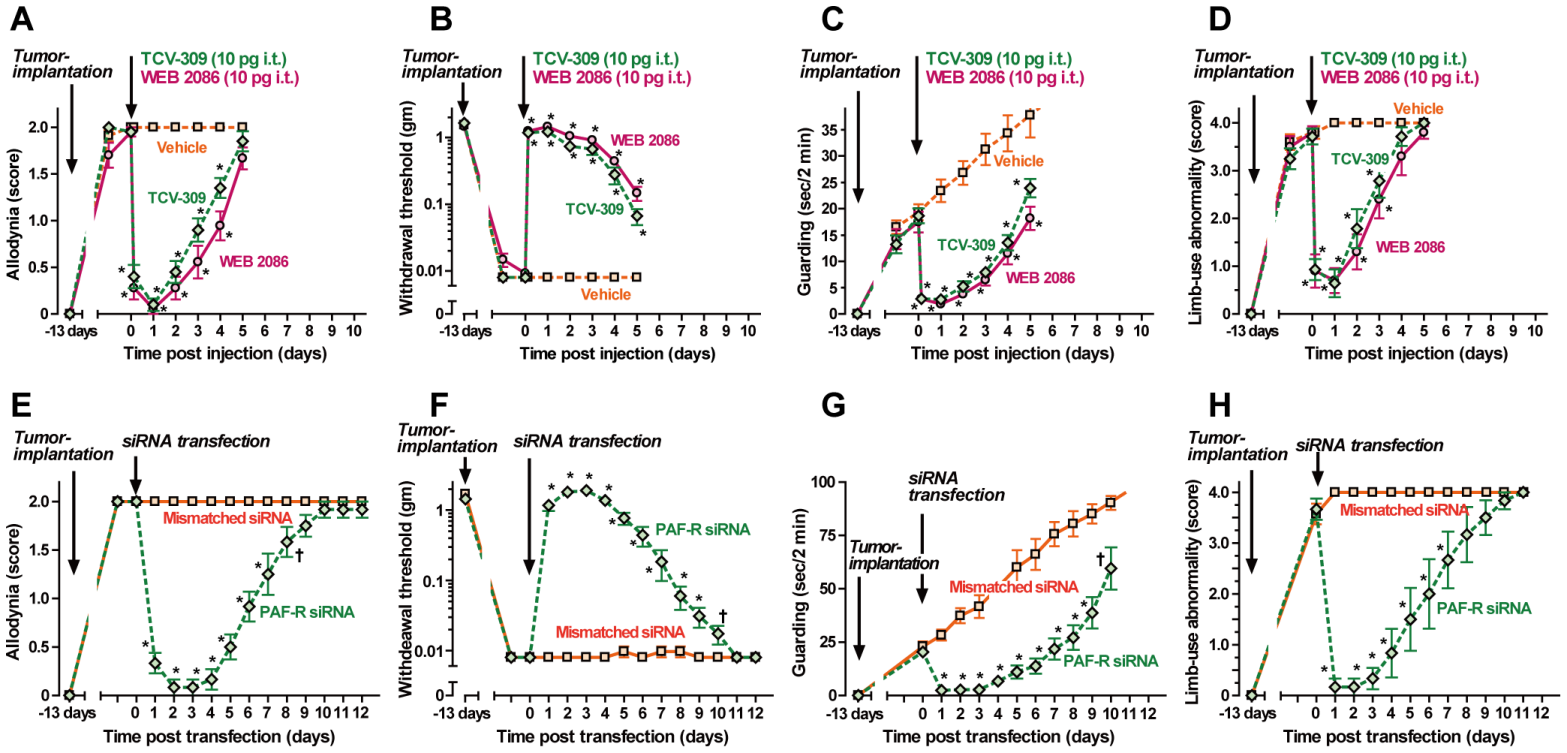
Effects of blockade of PAF receptors by intrathecal injection of PAF receptor antagonists (A–D) or knockdown of spinal PAF receptors by siRNA (E–H) on tactile allodynia (A, B, E, F), guarding behavior (C, G) and limb-use abnormality (D, H) in FBC mice. (A–D); Tumor cells were implanted into the intramedulla of left femur bone 13 days before the intrathecal (i.t.) injection of PAF receptor antagonists. TCV-309 (10 pg/mouse), WEB 2086 (10 pg/mouse) or the vehicle were injected intrathecally at time “0”. Data are expressed as the mean ± SEM., n = 8–12 mice per group. *P<0.01 compared with the corresponding control (vehicle treated) values, as determined by analysis of variance followed by Tukey-Kramer test. Control mice received injections with a vehicle: ACSF. The pain-related behaviors that developed after tumor implantation in mice were not affected by the vehicle treatments. (E–H); siRNA or mismatched siRNA of PAF-receptor mRNA were transfected into the spinal cord 13 days after tumor implantation. Data are expressed as the mean ± SEM. n = 8–10 mice per group. †P<0.05, *P<0.01 compared with the corresponding control (mismatched siRNA transfection) values, as determined by analysis of valiance followed by an unpaired Student's *t*-test. Control mice received injections with mismatched siRNA or a vehicle: HVJ-envelope only. The pain-related behaviors that developed after tumor implantation in mice were not affected by mismatched siRNA or vehicle treatments.

To confirm the site of cancer pain palliation by PAF antagonists, the effects of intrathecal injection of TCV-309 and WEB 2086, and also interference in the expression of spinal PAF receptors using siRNA of PAF receptor mRNA were examined. Spinal administration of 10 pg of these drugs 13 days post-tumor implantation produced rapid and marked suppression of tactile allodynia with the maximal effect at 1 day post-implantation and significantly suppressed the allodynia within 4 days (Figure 2A). Pain release effects evaluated by withdrawal threshold, guarding behavior and limb-use abnormality of intrathecal injection of TCV-309 and WEB 2086 were also achieved (Figure 2B–2D).

**Figure 3:**
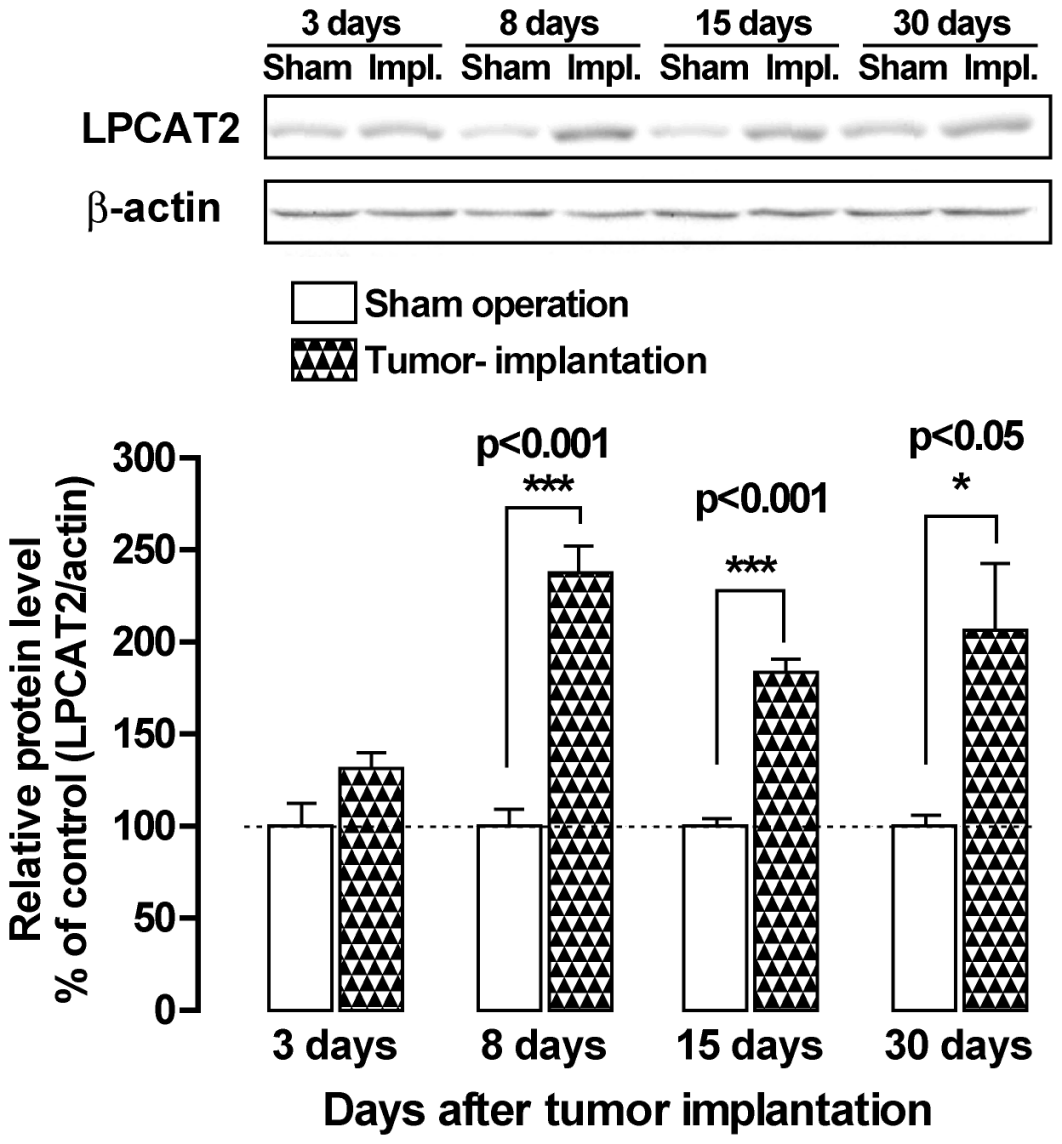
Changes in lysophosphatidylcholinr acyltransferase 2 (LPCAT2), an inducible PAF synthesis enzyume, in the spinal cords of mice after implantation of tumor cells. Alteration of spinal LPCAT2 expression 3, 8, 15, and 30 days after the levels of immunoreactivity were normalized to that of β-actin and represented as % induction compared with the values of sham-operated (deaden cells implanted) mice (mean ± SEM., n = 5–7). *P<0.05, ***P<0.001 versus the corresponding values in sham-operated mice (unpaired Student's *t*-test).

Knockdown of the expression of spinal PAF receptor protein was achieved by intrathecal transfer of PAF receptor siRNA in mice. As previously reported, a significant reduction in PAF receptor expression in normal mice and mice 15 days after surgery on the sciatic nerve, reaching 37.5±5.4% and 29.9±4.9% of the control at 3 days after siRNA transfection, respectively, while the expression was not altered by the HVJ-E vector alone or mismatched siRNA [5]. By siRNA transfection 13 days post-tumor implantation, the allodynia score, withdrawal threshold, guarding behavior an limb-use abnormality were improved with the peak effect at 2 to 3 days after siRNA transfection, while the pain-relief action gradually disappeared over 9 days (Figure 3E–3H). Injection of mismatched siRNA had no effect on the development of allodynia and pain behaviors. The amount of LPCAT2 protein significantly increased in the spinal cord at 8, 15 and 30 days after transplantation of NCTC 2472 tumor cells into mice tibia (Figure 3). The results support the concept that PAF increase in the spinal cord of tumor-bearing mice may relate to pain production and that the site of pain-relief action of the PAF receptor antagonists involve the spinal cord in FBC mice.

### Potentiation of the Analgesic Effect by Combination of PAF Receptor Antagonists with Morphine

**Figure 4:**
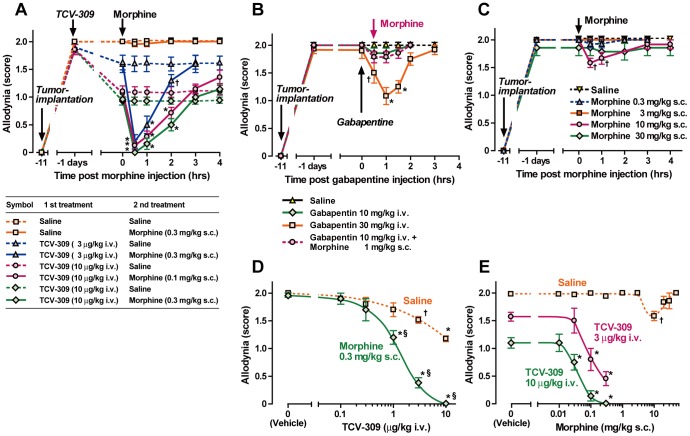
Enhanced pain reliving effect with a combination of TCV-309 and morphine in FBC mice. TCV-309, 3 and 10 μg/kg were injected i.v. at 10 days post tumor implantation and morphine 0.1 mg/kg and 0.3 mg/kg were injected s.c. 1 day after the injection of TCV-309 (A). Gabapentine 10 and 30 mg/kg i.v. were injected 11 days post tumor transplantation and morphine, 1 mg/kg was injected at 30 min after gabapentin injection (B). Morphine 0.3–30 mg/kg was injected at 11days post tumor transplantation (C). Pain-like behaviors were evaluated at 20 min after morphine injection. Various doses of TCV-309 were injected i.v. at 11 days post tumor implantation and morphine 0.3 mg/kg were injected s.c. 1 days after the injection of TCV-309 (D). One day after the injection of TCV-309, various doses of morphine were injected (E). Control mice received injections with a vehicle: saline. The pain-related behaviors that developed after tumor implantation in mice were not affected by vehicle treatments. Each group contained 11 mice. ^†^P<0.05, *P<0.01 compared with the corresponding control (vehicle treated) values, as determined by analysis of variance followed by Tukey-Kramer test. ^§^P<0.01 compared with the corresponding control (vehicle treated without morphine) values, as determined by analysis of variance followed by an unpaired Student's *t*-test.

Bone cancer pain is one of the most severe types of cancer pain and is often resistant to treatment even with opioid analgesics. Combinations of opioids and non-opioid analgesics are usual practice to obtain a better analgesic effect in clinical practice. Combined administration of morphine with TCV-309 was examined (Figure 4A). Morphine 0.1 and 0.3 mg/kg injection 1 day after TCV-309 3 and 10 μg/kg administration produced a marked reduction in the allodynia score at 30 min after morphine injection and the effect returned to each level of TCV-309 alone at 3 to 4 hrs after the administration. The combination of morphine 1 mg/kg with gabapentin 10 mg/kg i.v. had no significant anti-allodynia effect (Figure 4B). A large dose of gabapentin, 30 mg/kg, was required to produce a transient anti-allodynia effect. Morphine alone had little analgesic effect at 0.3 to 3 mg/kg, s.c. and a small effect at 10 mg/kg in the FBC model. A higher dose of 30 mg/kg of morphine was less effective (Figure 4C).

**Figure 5:**
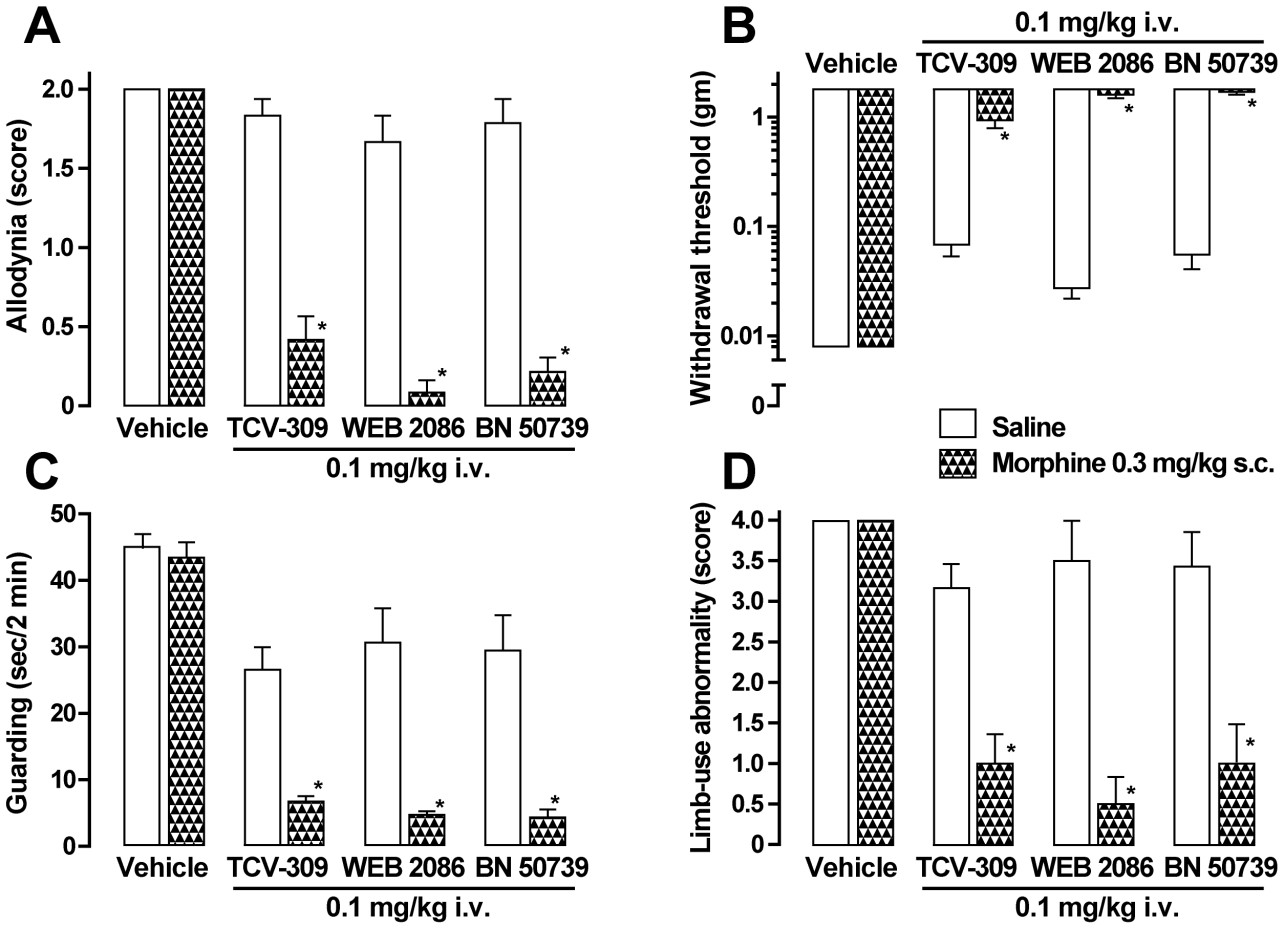
Enhanced pain reliving effect of TCV-309, WEB 2086, BN 50739 and morphine in FBC mice. TCV-309, WEB 2086 and BN 50739 were administered at 11 days post tumor implantation. Morphine 0.3 mg/kg s.c. was injected at 8 days after the injection of PAF receptor antagonists. Allodynia (A, B), guarding behavior (C) and limb-use abnormality (D) were evaluated at 20 min after the injection of morphine. Values represent the mean ± SEM. n = 11 mice per group. *P<0.01 compared with the corresponding control values, as determined by analysis of variance followed by an unpaired Student's *t*-test.

The dose-response relation of TCV-309 and morphine in combination is shown in Figure 4D and Figure 4E. Morphine 0.3 mg/kg and TCV-309 0.1–1.0 μg/kg at each dose alone had no anti-allodynia effect, but in combination, the allodynia score decreased by more than 90% depending on the dose of TCV-309 (Figure 4D). Morphine in combination with TCV-309 10 μg/kg as low as 0.03 mg/kg produced a significant anti-allodynia effect and abolished the allodynia response at 0.3 mg/kg (Figure 4E). Eight days after the injection of TCV-309, WEB 2086 and BN 50739, pain relieving effect of these compounds became not significant (Figure 5). However, the additional administration of morphine 0.3 mg/kg still markedly ameliorated allodynia and pain behaviors (Figure 5). These results showed that PAF receptor antagonists achieves long-lasting potentiation of the analgesic effect when morphine was administered.

### Effect of PAF Receptor Antagonists on Morphine-induced Constipation

**Figure 6:**
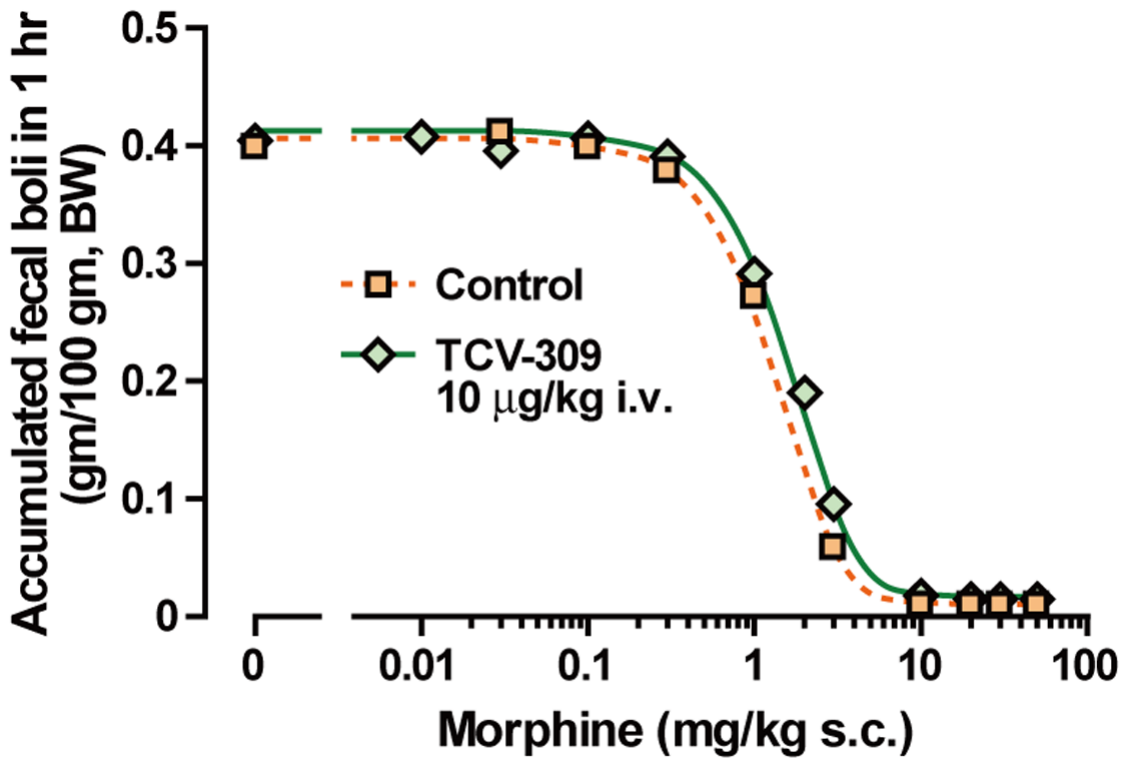
Morphine-induced constipation with or without TCV-309. Normal mice received with various doses of morphine and the accumulated feces on the floor over 60-309 was injected 1 day before morphine administration. Data are expressed as the mean. n = 10 mice per group.

Constipation is one of the most common side-effects of morphine and it appears in almost all patients treated with morphine. If the obstacle effect of morphine on intestinal activity is not potentiated, this side effect of morphine could be reduced by reducing the dosage of morphine without disturbing the analgesic effect. Morphine reduced the amount of feces from 1 mg/kg s.c. and abolished it more than 10 mg/kg. The dose-dependent constipation effect of morphine was not affected by TCV-309 10 μg/kg (Figure 6).

### Repeated Administration of TCV-309 Protected Against the Appearance of Pain-like Behavior

**Figure 7:**
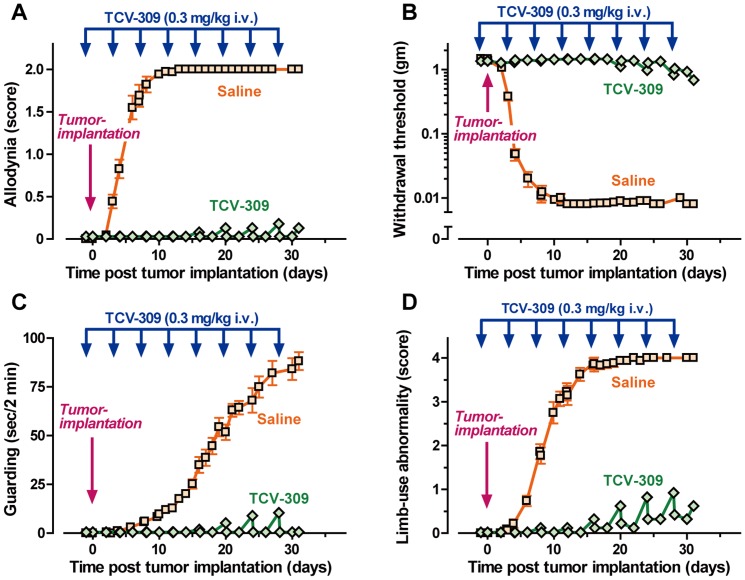
Effect of the repeated administration of TCV-309 on the pain-like behaviors in FBC mice. The administration of TCV-309 0.3 mg/kg i.v. was started 6 hr before the tumor implantation, given once a day and continued every 4 days up to 28 days. Allodynia (A, B), guarding behavior (C) and limb-use abnormality (D) were evaluated at 3 hr and 1, 2, 3 days after TCV-309 injection. Data are expressed as the mean ± SEM. n = 15 mice per group.

As the anti-allodynia effect of TCV-309 was long-lasting, the effect of the repeated administration was examined. In control mice, tactile allodynia appeared at 3 days after tumor implantation and reached to peak response at 8 days. Guarding behavior appeared at 10 days post tumor implantation and gradually increased over 30 days, while limb-use abnormality appeared at 8 days and reached a maximum at about 16 days post implantation (Figure 7A–7D). Injection of TCV-309 at 0.3 mg/kg, i.v. was started on the day mice were implanted with the tumor and was continued every 4 days for 28 days. Pain behaviors evaluated by allodynia score, withdrawal threshold, guarding behavior and limb-use abnormality did not appear during dosing (Figure 2). Thus, the treatment with TCV-309 before pain arose protected against crisis of pain and tolerance to TCV-309 analgesia did not develop.

### Treatment with TCV-309 Prolonged the Survival of FBC Mice

**Figure 8:**
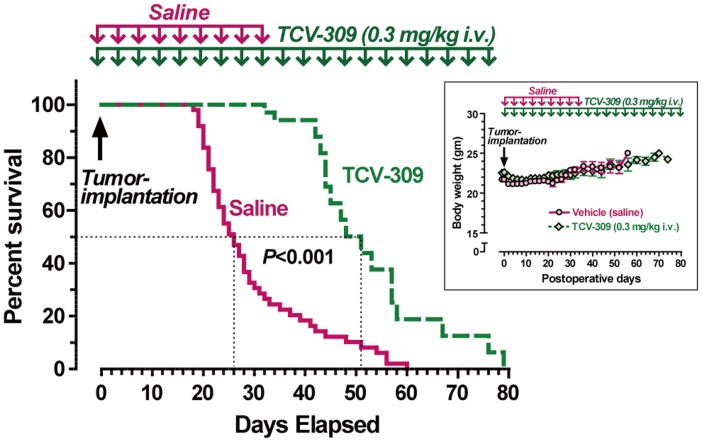
The Kaplan-Mayer survival curve of FBC mice and the change of body weight (insert). For the survival experiments, TCV-309 and saline were given once a day and continued every 4 days until the animals died (n = 17 and 50, respectively). Control mice received saline for 32 days. Days for 50% of mice died after receiving TCV-309 were significantly prolonged compared to the saline-treated control, P<0.001. Statistical analysis was performed by log-rank and Gehan–Breslow–Wilcoxon tests.

The effect of treatment with TCV-309 was evaluated in FBC mice (Figure 8). Median survival was significantly prolonged by repeated treatment with TCV-309; about 50% of FBC mice receiving saline died up to 26 days after tumor implantation, while about 50% of FBC mice receiving TCV-309 at 0.3 mg/kg every 4 days died up to 50 days. Gain of body weight by tumor-bearing mice was small during the observation period and the change in body weight was similar between control and TCV-308 treated mice (Figure 8 insert).

## Discussion

Bone cancer pain is often very complex; bone is highly innervated with C fibers, which are triggered by an inflammatory infiltrate secondary to cancer cells and others including acids, cytokine, growth factors, etc along with primary afferent destruction following osteoclast activation. Additionally, bone resorption weakens the bone under torsion, thus exciting mechanosensitive fibers within mineralized bone. The most intractable pain is often neuropathic in origin. However, in bone cancer pain, there is a unique neurochemical reorganization of the spinal cord, as well peripheral sensitization of afferent fibers innervating the cancerous bone, while spinal synaptic transmission mediated through Aδ and C fibers is enhanced in the substantia gelationsa across a wide area of lumbar levels following sarcoma implantation in the femur [12]. As the disease progresses, analgesics effective to treat inflammatory or neuropathic pain, even opioids, are frequently insufficient in this pain state [13], [14]. It has been reported that toll-like receptor (TLR) 4, which plays an important role in glial activation in neuropathic pain increased in the spinal expression of a rat model of cancer-induced bone pain and intrathecal injection of TLR4 siRNA or TLR4 signaling pathway blockers led to a pain relieving effect at an early stage, but not at day 16 of cancer-induced bone pain [15]. The present study demonstrated that intravenous administration of PAF receptor antagonists, TCV-309, BN 50739 and WEB 2086, effectively ameliorated allodynia and pain behaviors such as guarding and limb-use abnormalities in FBC mice. The pain relieving effects of PAF receptor antagonists are long lasting. We have previously suggested that the anti-allodynia effect of PAF antagonists in sciatic nerve injured mice is at least in part mediated by spinal relief of PAF-induced dysfunction of GlyRα3 [8]. In agreement with this concept, the present results showed that the intrathecal introduction of siRNA of PAF receptor mRNA effectively improved bone cancer pain behaviors in FBC mice. DRG contains a PAF synthesis enzyme, LPCAT2, and PAF receptor mRNA was increased in the ipsilateral DRG after nerve injury [6]. LPCAT2 mRNA and PAF receptor mRNA were increased in the spinal microglia after nerve injury in a rat spared nerve injury model [7]. Several studies have showed the ability of several cancer cell types to produce PAF and express PAF receptors on their membranes [16]–[20]. In the present study, the amount of LPCAT2 protein increased in the spinal cord of FBC mice, although in which cells the increase occurred remains to be elucidated. Therefore, PAF signaling in the microenvironment of the spinal pain transduction system may be increased by bone cancer due to peripheral nerve injury. We have further revealed a unique mode of action of TCV-309; TCV-309 is a specific competitive inhibitor of PAF receptors [21], but the potency of TCV-309 intensified as a function of time after administration, and the mode of action changed from a competitive manner within several hours after the injection of TCV-309 to a non-competitive manner later [8]. The intensification of the anti-allodynia potency of TCV-309 and change in its mode of action to a non-competitive manner as a function of time led us to speculate about a different mechanism of action; such as down-regulation of PAF receptors by binding TCV-309 to PAF receptors in the later stage of after nerve injury. This idea may explain the long lasting pain relieving effect of TCV-309.

The dose of morphine to block bone cancer pain was ten times that required to block inflammatory pain behaviors [12]. Morphine became less effective than oxycodone in FBC mice [22], [23]. Differential attenuation of μ-opioid receptor activation between morphine and oxycodone in FBC mice was reported, the former was significant whereas the latter was limited [24]. According with the evidence, morphine at 10 mg/kg s.c. had a tiny anti-allodynia effect in the present study, and the analgesic effect of a higher dose of 30 mg/kg of morphine was underestimated due to increased locomotion.

The essential finding in the present study is the enhanced pain reliving effect by combined administration of PAF receptor antagonists and morphine. For example, the combination of TCV-309 as low as 1 μg/kg i.v. and morphine 0.3 mg/kg s.c. (each drug had no pain relieving effect by itself) produced a significant anti-allodynia effect in FBC mice. Guarding behavior and limb-use abnormality were also reversed by the combined administration (data not shown). It is interesting that the enhanced pain relieving effect of PAF receptor antagonists, TCV-309, BN 50739 and WEB 2086 on morphine still remained at 8 days after the administration of these PAF receptor antagonists.

Constipation resulting from treatment with opioids is the most common component of a more general condition – opioid-induced bowel dysfunction [25]. Tolerance commonly develops to opioids during long-term use, requiring increased doses to achieve the same analgesia. Although some tolerance develops against the effects of opioids on gastrointestinal motility, constipation often persists unless remedial measures are taken [26]. Using lower doses of opioids will not prevent constipation because the dose that produces constipation is approximately 4-fold less than the analgesic dose [25], [27]. Therefore, some patients may discontinue opioids to avoid constipation. The present study showed that while a dose of morphine 10 mg/kg s.c. exhibited only a tiny pain relieving effect, 0.1 and 0.3 mg/kg of morphine in combination with PAF receptor antagonists produced a significant pain relieving effect. This result suggests that the combination use of morphine with PAF receptor antagonists may reduce the effective dose of morphine needed to below constipating dose. Activation of μ-receptors by morphine can have any of several effects depending on receptor location. Mu-opioid receptors in the central nervous system modulate pain perception and can depress respiratory function, while those in the gastrointestinal tract reduce bowel motility [26]. If μ-receptor-mediated signaling in the gastrointestinal tract was enhanced by PAF receptor antagonists, it would make defecation more difficult. The dose-response curve of morphine-induced defecation was not affected at all by 0.3 mg/kg of TCV-309, which is the dose producing the maximal analgesic effect and thus the possibility to avoid the appearance of morphine-induced constipation is suggested. On the other hand, PAF antagonists may cause side effects by interfering with the physiological roles of PAF, such as regulation of blood pressure, immunological or inflammatory responses, Ca^2+^ mobilization in polymorphonuclear leucocyte, or implantation of embryos, as shown by the creation of PAF receptor-transgenic and PAF receptor-deficient mice [28]. The possible side effects of PAF receptor antagonists could be reduced by combination with morphine. Therefore, good quality management of bone cancer pain could be achieved with a combination of morphine and PAF receptor antagonists. The long lasting effects of PAF receptor antagonists make it possible to relieve pain by repeated treatment at 4 day intervals and the results showed that these antagonists were effective from an early stage to a late stage, 30 days after tumor implantation at which point more than 50% of control mice died. The results further showed no formation of tolerance to repeated treatment with PAF receptor antagonists.

A role for PAF in tumor development has been suggested by the spontaneous development of skin tumors in transgenic mice overexpressing PAF receptors [29]. PAF also induced an autocrine proliferative loop in an endometrial cancer cell line HEC-1A [30], induced migration of Kaposi's cells [31], promoted migration and proliferation of tumor cells and neo-angiogenesis [17], acted as a promoter of melanoma metastasis [32], while PAF receptor-dependent pathways control tumor growth [33]. In addition, PAF receptor antagonists suppress cancer growth, proliferation and metastasis [20], [32]–[34]. The improvement in the survival of femoral bone tumor-bearing mice with TCV-309 may be due to its tumor suppressive action. As an another possibility, pain is an exquisite stressor and a cause for potential immune dysfunction, as shown in immunosuppression during the perioperative period, and thus poor pain control is assumed to promote tumor growth. Lilleme et al. [35] reported that patients with preexisting pain who received chemical splanchnicectomy with alcohol showed a significant improvement in survival. The authors reported that the achievement of better pain control with chemical splanchnicectomy may prolong life. Whether the improvement in survival by PAF receptor antagonist may include its indirect effect via suppression of pain is remained to be clarified.

Although the possibility could not be entirely ruled out that the tumor suppressive action of PAF antagonists may partly participate in the pain relieving effect of the repeated treatment of PAF antagonists in the FBC model, the acute pain relieving effect of PAF antagonists may be independent from anti-tumor action because the effect developed shortly after the intravenous injection and even by intrathecal injection.

Opiates in which their mechanisms of analgesia include the enhancement of the descending inhibitory pathway connected to the inhibitory inter-neurons such as glycinergic and GABAergic neurons. Recent findings emphasize that a reduction in the GABA_A_ receptor– and glycine receptor-mediated synaptic inhibition; ie., disinihibition of inhibitory neurotransmission within the dorsal horn, is implicated in the generation of neuropathic pain and may be a cause for insufficiency of morphine analgesia. Thus, reinforcement of the glycinergic neurotransmission by glycine transporter inhibitors are proposed as a novel drug discovery strategy for neuropathic pain [36]. Taking that PAF via an increase in nitric oxide/cyclic GMP cascade reduces GlyRα3 function in the spinal cord [5], the combination of PAF receptor antagonists and opioids, the former protects from disfunction of PAF-induced inhibitory neurotransmission and the latter enhances descending inhibitory pathway may represent a new strategy for the treatment of persistent cancer pain and the quality/quantity of life of patients.

## References

[1] Mundy GR (2002) Metastasis to bone: causes, consequences and therapeutic opportunities. Nat Rev Cancer 2: : 584–593. 10.1038/nrc867 12154351

[2] Portenoy RK, Lesage P (1999) Management of cancer pain. Lancet 353: : 1695–1700. 10.1016/S0140-6736(99)01310-0 10335806

[3] de Wit R, van Dam F, Loonstra S, Zandbelt L, van Buuren A, et al (2001) The amsterdam pain management index compared to eight frequently used outcome measures to evaluate the adequacy of pain treatment in cancer patients with chronic pain. Pain 91: 339–349. 11275392 10.1016/S0304-3959(00)00455-3

[4] Morita K, Morioka N, Abdin J, Kitayama S, Nakata Y, et al (2004) Development of tactile allodynia and thermal hyperalgesia by intrathecally administered platelet-activating factor in mice. Pain 111: 351–359. 15363879 10.1016/j.pain.2004.07.016

[5] Morita K, Kitayama T, Morioka N, Dohi T (2008) Glycinergic mediation of tactile allodynia induced by platelet-activating factor (PAF) through glutamate-NO-cyclic GMP signalling in spinal cord in mice. Pain 138: 525–536. 18353555 10.1016/j.pain.2008.01.030

[6] Hasegawa S, Kohro Y, Shiratori M, Ishii S, Shimizu T, et al. (2010) Role of PAF receptor in proinflammatory cytokine expression in the dorsal root ganglion and tactile allodynia in a rodent model of neuropathic pain. PLoS One 5 , e10467. 10.1371/journal.pone.0010467 PMC2862737 20454616

[7] Okubo M, Yamanaka H, Kobayashi K, Kanda H, Dai Y, et al (2012) Up-regulation of Platelet-activating factor synthases and its receptor in spinal cord contribute to development of neuropathic pain following peripheral nerve injury. Mol Pain 8: 8. 22296727 10.1186/1744-8069-8-8 PMC3293010

[8] Motoyama N, Morita K, Kitayama T, Shiraishi S, Uezono Y, et al (2013) Pain-releasing action of Platelet-activating factor (PAF) antagonists in neuropathic pain animal models and the mechanisms of action. Eur J Pain 17: 1156–1167. 23355413 10.1002/j.1532-2149.2013.00289.x

[9] Honore P, Mantyh PW (2000) Bone cancer pain: from mechanism to model to therapy. Pain Med 1: 303–309. 15101876 10.1046/j.1526-4637.2000.00047.x

[10] Luger NM, Sabino MA, Schwei ML, Mach DB, Pomonis JD, et al (2002) Efficacy of systemic morphine suggests a fundamental difference in the mechanisms that generate bone cancer vs inflammatory pain. Pain 99: 397–406. 12406514 10.1016/S0304-3959(02)00102-1

[11] Minami T, Nishihara I, Ito S, Sakamoto K, Hyodo M, et al (1995) Nitric oxide mediates allodynia induced by intrathecal administration of prostaglandin E_2_ or prostaglandin F_2α_ in conscious mice. Pain 61: 285–290. 7659439 10.1016/0304-3959(94)00183-F

[12] Yanagisawa Y, Furue H, Kawamata T, Uta D, Yamamoto J, et al (2010) Bone cancer induces a unique central sensitization through synaptic changes in a wide area of the spinal cord. Mol Pain 6: 38. 20602757 10.1186/1744-8069-6-38 PMC3020802

[13] Portenoy RK, Rayne D, Jacobsen P (1999) Breakthrough pain: characteristics and impact in patients with cancer pain. Pain 81: 129–134. 10353500 10.1016/s0304-3959(99)00006-8

[14] Becker R, Jakob D, Uhle EI, Riegel T, Bertalanffy H (2000) The significance of intrathecal opioid therapy for the treatment of neuropathic cancer pain conditions. Stereotact Funct Neurosug 75: 16–26. 10.1159/000048379 11416261

[15] Li X, Wang X-W, Feng X-M, Zhou W-J, Wang Y-Q, et al.. (2013) Stage-dependent anti-allodynia effects of intrathecal Toll-like receptor 4 antagonists in a rat model of cancer induced bone pain. J Physiol Sci DOI 10.1007/s12576-D12-0244-5. 10.1007/s12576-012-0244-5 PMC10717038 23392901

[16] Bussolino F, Arese M, Montrucchio G, Barrs L, Primo L, et al (1995) Platelet-activating factor produced in vitro by Kaposi's Sarcoma cells induces and Sustains in vivo angiogenesis. J Clin Invest 96: 940–952. 7543496 10.1172/JCI118142 PMC185282

[17] Bussolati B, Biancone L, Cassoni P, Russo S, Rola-Pleszczynski M, et al (2000) PAF produced by human brest cancer cells promotes migration and proliferation of tumor cells and neo-angiogenesis. Am J Pathol 157: 1713–1725. 11073830 10.1016/S0002-9440(10)64808-0 PMC1885724

[18] Bussolati B, Russo S, Deambrosis I, Cantaluppi V, Volpe A, et al (2002) Expression of CD154 on renal cell carcinomas and effect on cell proliferation, motility and platelet-activating factor synthesis. Int J Cancer 100: 654–661. 12209602 10.1002/ijc.10545

[19] Fallani A, Grieco B, Barletta E, Mugnai G, Giorgi G, et al (2002) Synthesis of platelet-activating factor (PAF) in transformed cell lines of a different origin. Prostaglandins Other Lipid Mediat 70: 209–226. 12428690 10.1016/s0090-6980(02)00109-0

[20] Tsoupras AB, Iatrou C, Frangia C, Demopoulos CA (2009) The implication of platelet-activating factor in cancer growth and metastasis: Potent beneficial role of PAF-inhibitors and antioxidants. Infectious Disorders-Drug Targets 9: 390–399. 19689381 10.2174/187152609788922555

[21] Terashita Z-I, Kawamura M, Takatani M, Tsushima S, Imura Y, et al (1992) Beneficial effects of TCV-309, a novel potent and selective platelet-activating factor antagonist in endotoxin and anaphylaxis shock in rodents. J Pharmacol Exp Ther 260: 748–755. 1738121

[22] Narita M, Nakamura A, Ozaki M, Imai S, Miyoshi K, et al (2008) Comparative Pharmacological profiles of morphine and oxycodone under a neuropathic pain-like state in mice: evidence for less sensitivity to morphine. Neuropsychopharmacology 33: 1097–1112. 17593930 10.1038/sj.npp.1301471

[23] Minami K, Hasegawa M, Ito H, Nakamura A, Tomii T, et al (2009) Morphine, oxycodone, and fentanyl exhibit different analgesic profiles in mouse pain models. J Pharmacol Sci 111: 60–72. 19729873 10.1254/jphs.09139fp

[24] Nakamura A, Hasegawa M, Minami K, Kanbara T, Tomii T, et al (2013) Differential activation of the μ-opioid receptor by oxycodone and morphine in pain-related brain regions in a bone cancer pain model. Br J Pharmacol 168: 375–388. 22889192 10.1111/j.1476-5381.2012.02139.x PMC3572564

[25] Yuan C-S (2005) Handbook of opioid bowel syndrome. New York: Haworth Medical Press: Haworth Reference Press. 256 p.

[26] Akil H, Owens C, Gutstein H, Taylor L, Curran E, et al (1998) Endogenous opioids: overview and current issues. Drug Alcohol Depend 51: 127–140. 9716935 10.1016/s0376-8716(98)00071-4

[27] Yuan C-S, Foss JF (2000) Antagonism of gastrointestinal opioid effects. Reg Anesth Pain Med 25: 639–642. 11097674 10.1053/rapm.2000.8658

[28] Honda Z, Ishii S, Shimizu T (2002) Platelet–activating factor receptor. J Biochem 131: 773–779. 12038971 10.1093/oxfordjournals.jbchem.a003164

[29] Ishii S, Nagase T, Tashiro F, Ikuta K, Sato S, et al (1997) Bronchial hyperreactivity, increased endotoxin lethality and melanocytic tumorigenesis in transgenic mice overexpressing platelet-activating factor receptor. EMBO J 16: 133–142. 9009274 10.1093/emboj/16.1.133 PMC1169620

[30] Maggi M, Bonaccorsi L, Finetti G, Carioni V, Muratori M, et al (1994) Platelet-activating factor mediates an autocrine proliferative loop in the endometrial adenocarcinoma cell line HEC-1A. Cancer Res 54: 4777–4784. 7520361

[31] Biancone L, Cantaluppi V, Boccellino M, Bussolati B, Del Sorbo L, et al (1999) Motility induced by human immunodeficiency virus-1 Tat on Kaposi's sarcoma cells requires platelet-activating factor synthesis. Am J Pathol 155: 1731–1739. 10550329 10.1016/S0002-9440(10)65488-0 PMC1866979

[32] Melnikova V, Bar-Eli M (2007) Inflammation and melanoma growth and metastasis: The role of platelet-activating factor (PAF) and its receptor. Cancer Metastasis Rev 26: 359–371. 17721743 10.1007/s10555-007-9092-9

[33] de Oliveira SI, Andrade LNS, Onuchic AC, Nonogaki S, Fernandes PD, et al (2010) Platelet-activating factor receptor (PAF-R)-dependent pathways control tumor growth and tumor response to chemotherapy. BMC Cancer 10: 200. 20465821 10.1186/1471-2407-10-200 PMC2881890

[34] Im SY, Ko HM, Kim JW, Lee HK, Ha TY, et al (1996) Augmentation of tumor metastases by platelet-activating factor. Cancer Res 56: 2662–2665. 8653713

[35] Lillemoe KD, Cameron JL, Kaufman HS, Yeo CJ, Pitt HA, et al (1993) Chemical splanchnicectomy in patients with unresectable pancreatic cancer. A prospective randomized trial. Annals Surgery 217: 447–457. 10.1097/00000658-199305010-00004 PMC1242819 7683868

[36] Dohi T, Morita K, Kitayama T, Motoyama N, Morioka N (2009) Glycine transporter inhibitors as a novel drug discovery strategy for neuropathic pain. Pharmacol Ther 123: 54–79. 19393690 10.1016/j.pharmthera.2009.03.018

